# From Luminal to Triple Negative: 3D Spheroids Reveal Molecular and Phenotypic Differences Across Breast Cancer Subtypes

**DOI:** 10.3390/ijms27083529

**Published:** 2026-04-15

**Authors:** Maria Miguel Castro, Letícia Maretti, Catarina Esquível, Bárbara Sousa, Carmen Jerónimo, Andrew J. Ewald, Joana Paredes

**Affiliations:** 1 Cancer Metastasis, i3S, Institute for Research and Innovation in Health, University of Porto, 4200-135 Porto, Portugal; mcastro@i3s.up.pt (M.M.C.); leticiamaretti@outlook.com (L.M.); cesquivel@i3s.up.pt (C.E.); bbeatrizs@gmail.com (B.S.); 2 Faculty of Medicine, University of Porto, 4200-319 Porto, Portugal; 3 Cancer Biology and Epigenetics Group, Research Center of IPO Porto (CI-IPOP)/CI-IPOP@RISE Health Research Network, Portuguese Oncology Institute of Porto (IPO Porto), 4200-072 Porto, Portugal; carmenjeronimo@ipoporto.min-saude.pt; 4 Porto Comprehensive Cancer Center Raquel Seruca (P.CCC Raquel Seruca), 4200-072 Porto, Portugal; 5 ICBAS, School of Medicine and Biomedical Sciences, 4050-313 Porto, Portugal; 6 Department of Cell Biology, Johns Hopkins University School of Medicine, Baltimore, MD 21205, USA; andrew.ewald@jhmi.edu; 7 Giovanis Institute for Translational Cell Biology, Johns Hopkins University School of Medicine, Baltimore, MD 21205, USA; 8 Cancer Invasion and Metastasis Program, Sidney Kimmel Comprehensive Cancer Center, Johns Hopkins University, Baltimore, MD 21231, USA

**Keywords:** breast cancer, molecular subtypes, 3D tumor spheroids, EMT

## Abstract

Breast cancer is classified into distinct molecular subtypes, including Luminal A, Luminal B, HER2-enriched, Basal-like, and Claudin-low. While traditional studies mostly use 2D cell cultures, 3D models better mimic in vivo tumor conditions. In this study, we generated and characterized 3D spheroids from breast cancer cell lines representing different molecular subtypes. Morphologically, spheroids were either compact (MCF-7/AZ, T47D, BT474, MDA-IBC-3, BT-20, SUM149PT) or loosely adhered (MDA-MB-468, SK-BR-3, MDA-MB-231), while retaining key parental subtype biomarkers. Cell viability decreased with increasing spheroid size, but apoptotic cCasp3 staining was restricted to Basal-like spheroids. Compact spheroids expressed E- and/or P-cadherin, indicating epithelial or epithelial–mesenchymal transition (EMT) hybrid traits, while loose spheroids showed vimentin expression linked to a mesenchymal phenotype. In conclusion, EMT-associated features, rather than intrinsic molecular subtype, may contribute to 3D spheroid architecture of breast cancer cell lines.

## 1. Introduction

Breast cancer is a significant global health challenge, recognized as the most prevalent malignancy worldwide, accounting for approximately 11.6% of all new cancer diagnoses annually [1], and remains the leading cause of cancer-related mortality among women worldwide. It is a highly heterogeneous disease that comprises several molecular subtypes and varying cellular morphologies, which significantly impact its prognosis and treatment strategies [2]. In recent decades, transcriptomic studies have established five intrinsic breast cancer molecular subtypes, Luminal A, Luminal B, HER2-enriched, Basal-like, and Claudin-low, each exhibiting distinct underlying biology, prognosis, and clinical behavior [3,4,5]. Luminal tumors usually express hormonal receptors (ER and PR) and respond to endocrine therapy, which are further classified into two subtypes: Luminal A and Luminal B. While Luminal A is characterized by high expression of ER and/or PR, lack of HER2 overexpression and low levels of Ki-67, Luminal B exhibits lower hormone receptor expression, may present HER2 positivity, and shows higher levels of Ki-67, being correlated with a more aggressive phenotype, therapy resistance and poor prognosis compared to Luminal A [6,7]. Conversely, HER2-enriched breast cancer is characterized by the overexpression of HER2, leading to aggressive tumor growth and metastasis [8,9]; however, HER2-targeted therapies have significantly improved these patients’ outcomes [10,11]. Basal-like tumors are predominantly triple-negative (ER, PR, and HER2-negative), displaying high proliferation rates, genetic instability, enrichment of basal mammary markers, and poor prognosis [12,13]. Finally, Claudin-low tumors are considered a distinct subtype characterized by low expression of claudins and tight junction cell–cell adhesion proteins, as well as by an enrichment of stem cell-like features, being also predominantly triple-negative breast cancers [14].

Two-dimensional (2D) monolayer culture systems are widely used in cancer research because of their low cost and simplicity. However, these 2D systems do not replicate in vivo conditions and the complexity of the tumor microenvironment. In vivo, tumor cells grow in a complex three-dimensional (3D) architecture, where gradients of oxygen, nutrients, and metabolites establish heterogeneous zones of proliferation, hypoxia, and necrosis [15]. Additionally, dynamic cell–cell and cell-extracellular matrix (ECM) interactions influence key cellular processes, such as cell differentiation, invasion, and drug resistance. These processes are incompletely modelled in 2D systems, which limits their translational relevance [15,16]. Diverse 3D culture systems have been developed, including spheroids, organoids, and scaffold-based models, to provide a more physiologically relevant platform for cancer research. Among them, tumor spheroids represent a robust, reproducible, and cost-effective approach that mimics the structural and functional characteristics of solid tumors [17,18]. Spheroids recapitulate essential features of tumor biology, such as the establishment of proliferation gradients, induction of hypoxia-mediated signalling, altered gene expression profiles, and reduced drug penetration [19]. Larger spheroids, above the critical size of 400–600 µm, sustain oxygen and nutrient gradients. The result is the formation of a central necrotic core, similar to that observed in poorly vascularized tumors, leading to the formation of three different layers of cells: proliferative cells on the outer layer, quiescent cells on the inner layer, and necrotic cells in the spheroids’ core [15,18,20]. However, even spheroids between 200 and 500 µm are sufficiently large to develop gradients of oxygen, nutrients, and catabolites [20,21]. We anticipate that breast cancer cell lines from different molecular subtypes should exhibit distinct capacities to form and maintain 3D spheroids, which would reflect variations in their biology, including the expression of different adhesion molecules, stem-like properties, and metabolic adaptation.

In this study, we generated and characterized tumor spheroids from breast cancer cell lines representing distinct molecular subtypes. By comparing spheroids’ morphology, growth kinetics, circularity, and viability, we aimed to identify subtype-specific features that may underlie differences in tumor behavior and therapy response. A better understanding of these differences may not only enhance the predictive value of 3D preclinical models but also guide the development of subtype-specific therapeutic strategies.

## 2. Results

### 2.1. Establishment and Characterization of Breast Cancer Tumor Spheroids

In vitro 3D breast cancer tumor spheroids were generated, using monocultures of nine breast cancer cell lines representing distinct molecular subtypes: MCF-7/AZ and T47D as Luminal A; BT474 as Luminal B; SK-BR-3 and MDA-IBC-3 as HER2-enriched; BT-20 and MDA-MB-468 as Basal-like; and MDA-MB-231 and SUM149PT as Claudin-low (Table 1). These molecular subtypes were determined based on previous studies performed by Neve et al. [22] and Prat A et al. [14,23]. To establish these spheroids, microwell array technology was used as previously described [24,25] (Figure S1). Briefly, cells were seeded in different numbers (1000, 2500, and 5000 cells per spheroid) into agarose micro-molds, where cell–surface interactions are minimized, due to non-adhesive and hydrophilic properties, leading to spheroid formation due to cell–cell adhesion cues.

### 2.2. Morphological Characterization of Breast Cancer Tumor Spheroids

**Figure 1:**
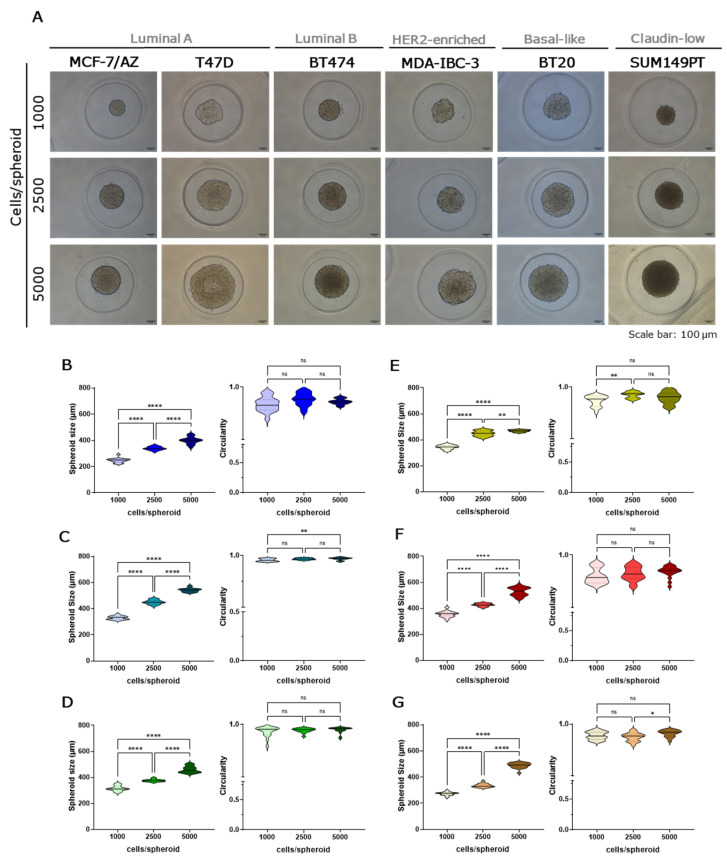
Compact spheroids generated by several breast cancer cell lines [MCF-7/AZ (blue), T47D (blue), BT474 (green), MDA-IBC-3 (yellow/green), BT20 (red) and SUM149PT (brown)] and seeded at three different cell densities (1000, 2500 and 5000 cells/spheroid). Brightfield microscopy images of spheroids’ morphology after 48 h. Scale bars represent 100 µm (**A**). Measurement of spheroids’ size and circularity for MCF-7/AZ (**B**), T47D (**C**), BT474 (**D**), MDA-IBC-3 (**E**), BT20 (**F**), and SUM149PT (**G**) using the Harmony high-content imaging and analysis software in three independent experiments. Data are shown as mean ± SD and the level of significance was set at * *p* < 0.05, ** *p* < 0.01, **** *p* < 0.0001, and ns, not significant (*p* ≥ 0.05).

Brightfield images were acquired and spheroids’ size and circularity (with 1.0 representing a perfect circle) were assessed. Morphological integrity was also evaluated by H&E staining. After 48 h in culture, distinct patterns of spheroids morphology were observed across the different cell lines tested. A compact and well-adherent spheroid was found for MCF-7/AZ, T47D, BT474, MDA-IBC-3, BT-20, and SUM149PT cell lines (Figure 1A). The spheroids’ size increased in a cell number-dependent manner and consistently presented high circularity values (approximately 1.0), indicative of their compact and circular morphology regardless of the initial cell number (Figure 1B–G). Additionally, H&E staining reinforced these observations, showing highly cohesive and compact cellular aggregates formed by these cell lines (Figure S2). It is noteworthy that, for the MDA-IBC-3 cell line, spheroids generated with 2500 cells presented a significant higher circularity compared to the ones formed with 1000 cells per spheroid (Figure 1E), while in SUM149PT, 5000 cells/spheroid showed increased circularity compared with 2500 cells/spheroid (Figure 1G).

**Figure 2:**
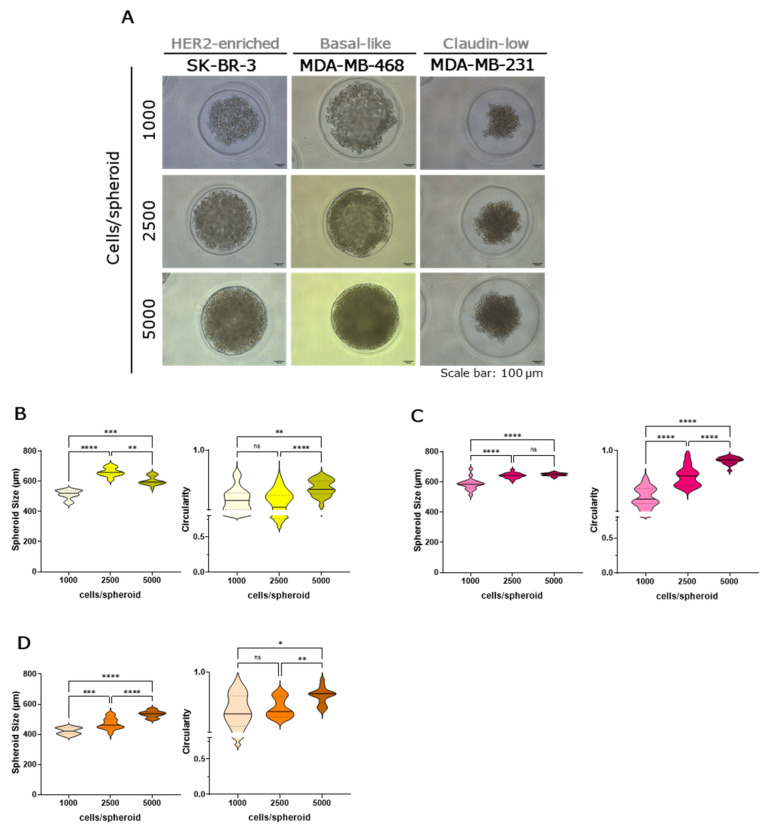
Loosely adhered spheroids generated by several breast cancer cell lines [SK-BR-3 (yellow), MDA-MB-468 (pink), and MDA-MB-231(orange)] and seeded in three different cell densities (1000, 2500 and 5000 cells/spheroid). Brightfield microscopy images of spheroids’ morphology after 48 h. Scale bars represent 100 µm (**A**). Measurement of spheroids’ size and circularity for SKBR-3 (**B**), MDA-MB-468 (**C**) and MDA-MB-231 (**D**) using the Harmony high-content imaging and analysis software in three independent experiments. Data are shown as mean ± SD and the level of significance was set at * *p* < 0.05, ** *p* < 0.01, *** *p* < 0.001, **** *p* < 0.0001 and ns, not significant (*p* ≥ 0.05).

Conversely, loosely adhered spheroids were found with SK-BR-3, MDA-MB-468, and MDA-MB-231 cell lines (Figure 2A). These spheroids showed lower circularity and size variability across seeding densities. Regarding the spheroids’ size, for the SK-BR3 cell line, the ones formed with 2500 cells were significantly larger than those formed with both 1000 and 5000 cells (Figure 2B). But, as expected, 5000 cells generated significantly larger spheroids than 1000 cells. In the MDA-MB-468 cell line, the spheroids’ size increased with seeding density; however, no statistically significant differences were observed between 2500 and 5000 cells per spheroid (Figure 2C). Less cohesive and circular aggregates were also confirmed by H&E staining, which also indicated no evidence of necrotic core in any spheroid across all tested seeding densities (Figure S2).

### 2.3. Molecular Characterization of Breast Cancer Tumor Spheroids

**Figure 3:**
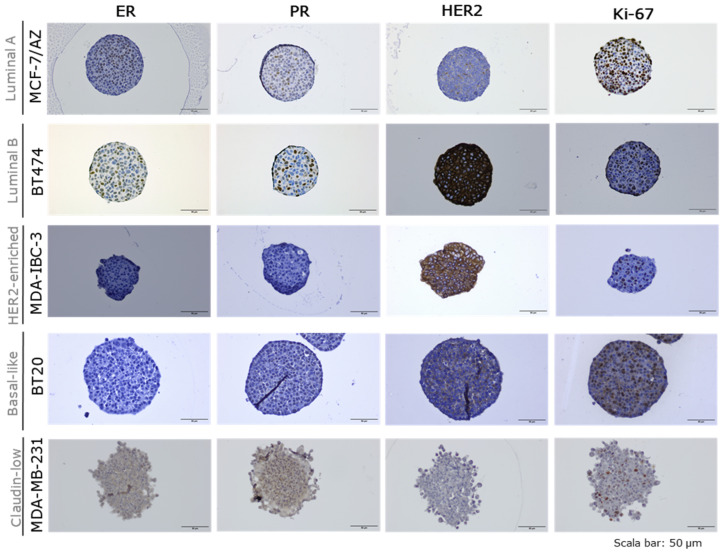
Representative images of each breast cancer molecular subtype (Luminal A-MCF-7/AZ; Luminal B-BT474; HER2-enriched–MDA-IBC-3; Basal-like-BT20; and Claudin-low–MDA-MB-231). Immunohistochemistry for hormonal receptors (ER-Estrogen; PR-Progesterone), human epidermal growth factor receptor 2 (HER-2) and Ki67 (proliferation marker) using breast cancer spheroids (2500 cells/spheroid). Scale bars represent 50 µm.

Breast cancer molecular subtypes were validated qualitatively through immunohistochemistry, by studying the expression of key markers, such as ER, PR, HER2, and Ki-67 (Figure 3 and Figure S3). As expected, Luminal A (MCF-7/AZ and T47D) spheroids were positive for both ER and PR. However, although MCF-7/AZ showed negativity for HER2, as expected, T47D spheroids showed HER2 positivity. Regarding Ki-67, MCF-7/AZ spheroids presented high expression for this marker, while the T47D ones showed lower levels; however, both demonstrated widespread nuclear staining throughout the spheroid core. For Luminal B (BT474) spheroids, a strong HER2 expression was found, as well as positivity for both ER and PR. An intermediate Ki-67 staining was observed, with a slightly more heterogeneous distribution. Concerning SK-BR-3 and MDA-IBC-3, both presented strong HER2 expression, as expected, and were negative for ER and PR. High Ki-67 expression was observed in SK-BR-3, whereas MDA-IBC-3 showed lower expression levels of this marker. In both cell lines, positive nuclei were dispersed throughout spheroid structures. As expected, spheroids derived from the Basal-like (BT-20 and MDA-MB-468) and Claudin-low (SUM149PT and MDA-MB-231) molecular subtypes were triple-negative for classical receptors (ER-, PR-, and HER2-). For Ki-67, high expression was observed in BT-20 and MDA-MB-468 spheroids, consistent with their known aggressive and fast-growing phenotype. Claudin-low spheroids displayed a lower proliferation index. MDA-MB-231 showed low and sparse Ki-67-positive nuclei, while SUM149PT presented an intermediate expression, mainly localized in the spheroids’ core.

### 2.4. Viability and Cell Death in Breast Cancer Tumor Spheroids

**Figure 4:**
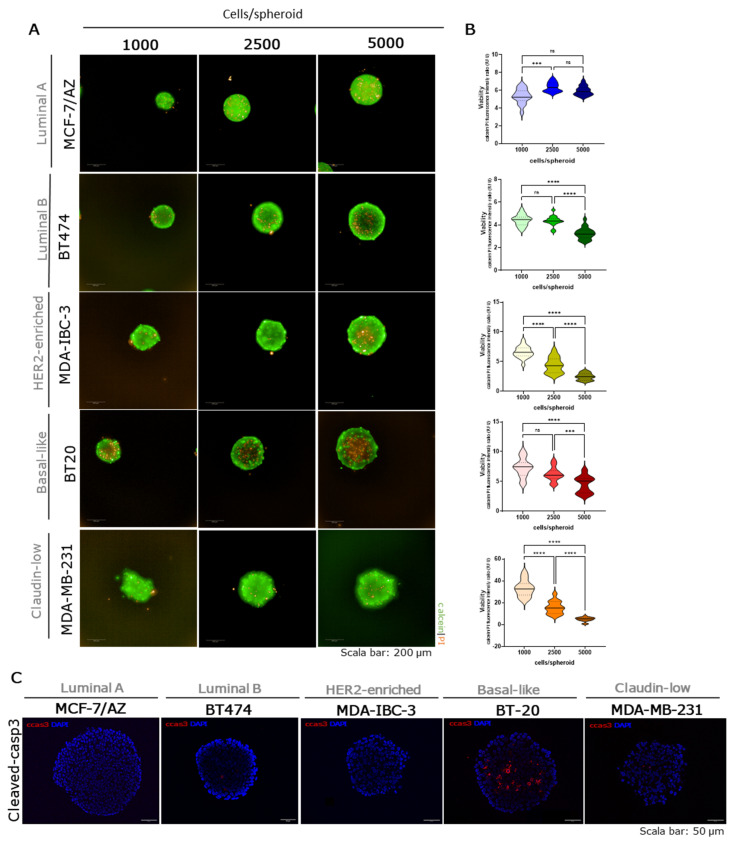
Evaluation of cell viability and cleaved-caspase 3 for each breast cancer molecular subtype: Luminal A-MCF-7/AZ (blue); Luminal B-BT474 (green); HER2-enriched–MDA-IBC-3 (yellow/green); Basal-like-BT20 (red); and Claudin-low–MDA-MB-231 (orange). Viability was evaluated using calcein/PI staining, where calcein (green) label live cells, and PI (red) the dead cells. Scale bars represent 200 µm (**A**). Quantification of the fluorescent intensity (RFU—relative fluorescent units) of calcein and PI ratio using the Harmony high-content imaging and analysis software in three independent experiments (**B**). Representative images of cleaved-caspase-3 (cCasp3, red) staining and nuclei counterstained with DAPI (blue) in the 2500 cells/spheroid after 48 h in suspension. Scale bars represent 50 µm (**C**). Data are shown as mean ± SD, and the level of significance was set at *** *p* < 0.001,**** *p* < 0.0001, and ns, not significant (*p* ≥ 0.05).

Cell viability was evaluated through a live/dead staining, using Calcein-AM and propidium iodide (PI) staining after 48 h in suspension (Figure 4 and Figure S4). SK-BR-3, MDA-IBC-3, MDA-MB-231, MDA-MB-468, and SUM149PT spheroids showed a statistically significant decrease in viability in a cell-number-dependent manner. Conversely, MCF-7/AZ spheroids, formed with 1000 cells, presented the lowest viability compared to 2500 and 5000 cells per spheroid. Moreover, 5000 cells per spheroid of T47D, BT474, and BT-20 showed the lowest viability; however, no statistically significant differences were observed between 1000 and 2500 cells per spheroid. In addition, cell death mechanisms were assessed using immunofluorescence staining for cCasp3, a well-established marker of apoptosis. Basal-like spheroids were the only ones exhibiting positive expression, mainly in the core, which suggests central apoptotic activity.

### 2.5. Epithelial-Mesenchymal Transition in Breast Cancer Tumor Spheroids

**Figure 5:**
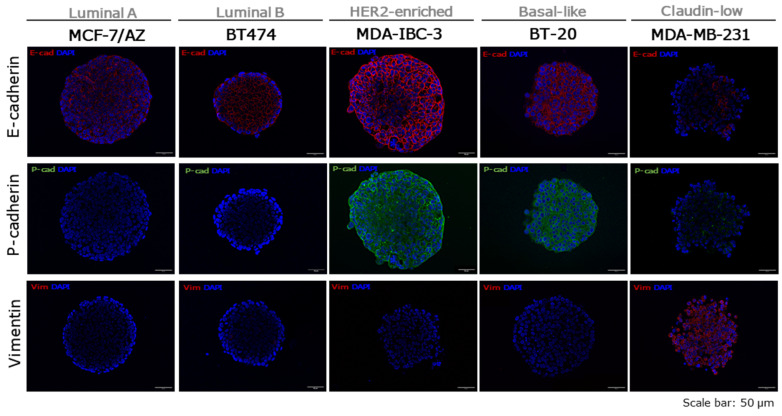
Representative images showing the expression of EMT markers for each breast cancer molecular subtype (Luminal A-MCF-7/AZ; Luminal B-BT474; HER2-enriched–MDA-IBC-3; Basal-like-BT20; and Claudin-low–MDA-MB-231). E-cadherin staining (red) was used as an epithelial marker, vimentin staining (red) as a mesenchymal marker, and E-cadherin (red) and P-cadherin (green) representing the hybrid EMT phenotype. All samples were counterstained with DAPI (blue). Scale bars represent 50 µm.

Epithelial–mesenchymal transition (EMT) is a biological process characterized by the conversion of epithelial cells into more invasive mesenchymal cells. This transition involves a complex reprogramming of gene expression, leading to the downregulation of epithelial markers, such as E-cadherin, and the upregulation of mesenchymal markers, including vimentin [26,27]. The co-expression of E- and P-cadherin has been established as a possible marker of a hybrid EMT phenotype [26,28,29,30,31]. Spheroids’ epithelial and mesenchymal phenotypes were qualitatively assessed using an immunofluorescence staining of epithelial (E-cadherin), mesenchymal (vimentin), and hybrid (E- and P-cadherin) markers (Figure 5 and Figure S5).

The results showed that spheroids representing the Luminal subtype (MCF-7/AZ, T47D, and BT474) exhibited strong membrane expression of E-cadherin and no expression of P-cadherin and vimentin, supporting the epithelial phenotype, as well as the strong compactness and cell–cell adhesion observed before. HER2-enriched SKBR3 spheroids showed vimentin expression at the membrane, but absence of both E- and P-cadherin, indicating a mesenchymal phenotype consistent with their loose adhesion. In contrast, MDA-IBC-3 spheroids revealed strong co-expression of both cadherins at the cell membrane, with no vimentin expression, demonstrating an epithelial phenotype and consistent adhesive behavior. Basal-like spheroids (MDA-MB-468 and BT-20) showed co-expression of both E- and P-cadherin. While no vimentin expression was observed in BT-20 spheroids, MDA-MB-468 spheroids showed slight vimentin expression. This result is consistent with the strong compactness of BT-20 spheroids, but also with the loose aggregation of MDA-MB-468 spheroids. In the Claudin-low subtype, MDA-MB-231 spheroids showed minimal E- and P-cadherin expression and strong vimentin expression, in accordance with their loose adhesion profile. Conversely, SUM149PT spheroids, similarly to MDA-IBC-3, showed a strong E- and P-cadherin co-expression and no vimentin, indicating a hybrid EMT phenotype consistent with the adhesion observed during spheroid formation. Supporting these observations, we performed a bioinformatic analysis using data from the Cancer Cell Line Encyclopedia (CCLE) to associate the observed 3D spheroid morphology with EMT scores obtained from the Molecular Signatures Database (MSigDB). The analysis indicated that cell lines forming loosely adherent spheroids tend to display higher EMT scores, consistent with a more mesenchymal phenotype, whereas cell lines forming compact spheroids show lower EMT scores, indicative of a more epithelial profile. Although this difference did not reach statistical significance, likely due to the limited number of cell lines analyzed, the results support our observation that epithelial-like cell lines preferentially form compact spheroids, while mesenchymal-like cell lines are associated with loosely adherent spheroid structures (Figure S6).

## 3. Discussion

Over the course of recent years, 3D models have become widely applied in cancer research, providing a valuable tool to evaluate drug response, tumor-specific growth patterns, and cell–cell interactions. These models provide a more physiologically relevant system for cancer research by more accurately mimicking in vivo architecture and tumor microenvironment than 2D models [15]. In this study, we successfully established and characterized 3D breast cancer tumor spheroids across nine cell lines representing distinct molecular subtypes.

Morphological analyses revealed differences in spheroid integrity and cohesion. Luminal A and B cell lines (MCF-7/AZ, T47D, BT474), as well as MDA-IBC-3, BT-20, and SUM149PT, formed compact, highly circular spheroids, reflecting strong cell-cell adhesion and enriched epithelial characteristics. Actually, T47D spheroids showed a lumen formation, which is a distinctive morphological feature that has been previously reported in the literature under different cell densities and incubation times [32,33]. In contrast, SK-BR-3, MDA-MB-468, and MDA-MB-231 produced loosely aggregated, irregular spheroids, indicative of a weaker cohesion and more mesenchymal-like traits. Our results are in accordance with the literature, which shows that SK-BR-3 and MDA-MB-468 presented a grape-like colony morphology in 3D cultures, while MDA-MB-231 displays a stellate structure [34]. Although MDA-MB-468 spheroids exhibited an increase in circularity (approaching 1.0) at the 5000 cells per spheroid condition, this does not reflect the formation of a truly compact spheroid, but rather a technical limitation of the micro-mold culture system. Agarose micro-molds are designed to support spheroid growth within a diameter range of approximately 300–800 µm. Under these conditions, however, MDA-MB-468 spheroids exceeded ~600 µm and approached the physical limits of the microwells. As a result, spatial confinement likely restricted normal spheroid expansion, and mechanical constraints imposed by the well walls artificially increased the measured circularity. Additionally, another study established that SK-BR-3 and MDA-MB-231 cells presented loose aggregates or single-cell suspension structures for spheroids generated using the liquid overlay technique [35]. The morphological differences observed were partly dependent on initial seeding density, with higher cell numbers generally promoting larger spheroids [25,36,37], although some cell lines displayed non-linear growth patterns, suggesting intrinsic regulation of cell-cell adhesion and proliferation. H&E staining corroborated these observations, highlighting cohesive cellular architecture in compact spheroids and loose organization in mesenchymal-like spheroids, with no necrotic cores observed across the 48 h culture period. One of the limitations of our study is the suspension period, since the extension of the culture period may alter these results, leading to increased necrosis in the spheroid core. Additionally, a cell organization and ultrastructure study could be performed to better understand the differences in spheroids over time of incubation.

Molecular characterization confirmed that 3D spheroids retained the key markers of their parental molecular subtypes [38,39,40,41]. Luminal spheroids were positive for ER and PR, with variable Ki-67 proliferation patterns; notably, MCF-7/AZ exhibited widespread high Ki-67 expression, which contrasts with prior reports showing low proliferative capacity of MCF-7 cells in 3D spheroids [42,43]. It is important to consider that in one of the studies the spheroids were performed in a scaffold-based method in collagen type I [42], and that in the other study, they used different cell line media [43], which can have an impact on the expression of biological markers [44]. T47D showed some Ki-67 peripheral localization, suggesting potential gradients in proliferation even in the absence of necrosis. Additionally, T47D showed HER2 expression in 3D spheroids, even though this cell line has already been described as HER2 negative in 2D cultures. However, one study shows that T47D long-term treated with an anti-estrogen drug can resist and shift from ER+/HER2- to HER2+/Src+, with a significant increase in HER2 expression and Src activity [45]. Moreover, an increase in fibronectin was already found in MDA-MB-231, which increases the activity of Src [46], and it was already reported that T47D 3D models show an increase in fibronectin mRNA levels expression [47]. Thus, our data suggest the hypothesis that T47D grown in 3D can have more fibronectin and adhesion molecules expression, like FAK, which can increase Src activation and consequently HER2 activation in spheroids. However, this hypothesis requires additional validation, since we only assessed qualitatively. HER2-enriched spheroids demonstrated expected HER2 expression, but varied in proliferation, with SK-BR-3 showing high Ki-67 and MDA-IBC-3 exhibiting lower, more heterogeneous proliferation, highlighting intra-subtype heterogeneity. Basal-like and Claudin-low spheroids maintained their triple-negative status, with Basal-like cell lines showing high Ki-67 and central apoptosis, consistent with aggressive, rapidly proliferating phenotypes [48]. Claudin-low spheroids, particularly MDA-MB-231, displayed lower proliferation rates, reflecting the mesenchymal/stem-like phenotype characteristic of this subtype [14], where cells exhibit increased invasive capacity, rather than proliferative competence.

Cell viability analyses further emphasized molecular subtype-dependent behaviors. Viability decreased with increasing spheroid size in several cell lines, particularly in SK-BR-3, MDA-IBC-3, MDA-MB-231, MDA-MB-468, and SUM149PT, suggesting limitations in nutrient diffusion or accumulation of apoptotic signals [25,36,37].

Overall, the viability results indicate that compact spheroids exhibit increased cell death in the core and lower overall viability compared to loosely adherent spheroids, in which dead cells are more dispersed throughout the structure rather than concentrated in the center. Previous studies suggest that compact spheroids consume oxygen at a higher rate than loosely adherent ones and develop steeper hypoxic gradients from the surface to the core, indicating that oxygen distribution is closely related to spheroid compactness [49]. Additionally, Zanoni et al. reported that variations in spheroid shape (spherical versus non-spherical) can influence cell viability. The authors hypothesized that non-spherical spheroids may contain a greater proportion of viable cells because the reduced distance between individual cells and the culture medium interface increases the area available for active proliferation [50]. However, in the MCF-7/AZ cell line, cell viability did not decrease in the largest spheroids, unlike what was observed for the other cell lines. One possible explanation is that the size of these spheroids remained below 400 µm. According to the literature, oxygen and nutrient diffusion are more efficient in smaller spheroids, preventing the formation of severe hypoxic or necrotic regions [15,20]. Furthermore, previous studies have shown that 48 h in 3D culture may be insufficient to significantly reduce cell viability in MCF-7 spheroids generated using different 3D culture systems [51,52]. Therefore, it is possible that longer culture periods would lead to the development of a necrotic core in these spheroids, similar to what has been reported for other cell lines under comparable conditions. cCasp3 expression was restricted to Basal-like spheroids, mainly in the core, indicating central apoptosis as a feature of this aggressive molecular subtype [53]. Qualitative assessment of EMT markers revealed a strong correlation between EMT phenotype and spheroid morphology [38,46], although with notable exceptions. Luminal spheroids expressed E-cadherin and lacked P-cadherin and vimentin, consistent with their epithelial phenotype and compact morphology. SK-BR-3 and MDA-MB-231 spheroids presented a mesenchymal phenotype (vimentin positive), aligning with their loose adhesion, whereas MDA-IBC-3 and SUM149PT exhibited a hybrid EMT phenotype, with co-expression of E- and P-cadherin, yet retaining compact morphology, suggesting that partial EMT states may support both cell-cell adhesion and invasive potential [29]. Basal-like cell lines (BT-20 and MDA-MB-468) also showed E- and P-cadherin co-expression, but morphological outcomes varied, implying additional factors contributing to spheroid cohesion. Notably, it has been demonstrated that α-catenin exons 4 and 5 are homozygously deleted from the genome of MDA-MB-468 cells, leading to two aberrant transcript lengths, which contribute to their loose aggregation and vimentin expression, besides cadherins expression [54]. Overall, EMT-associated features, rather than molecular subtype, seem to contribute to spheroid’s morphology, with the mesenchymal phenotype (vimentin-positive), exhibiting loosely aggregated spheroids, and the epithelial-hybrid phenotype (E-cadherin and P-cadherin positive) characterized by a more compact organization.

In brief, our findings highlight the capacity of 3D spheroid models to recapitulate the intrinsic heterogeneity of breast cancer subtypes, encompassing differences in morphology, proliferation, EMT status, and viability. Although numerous studies already exist in the literature employing breast cancer spheroids, most of them only use one or two cell lines [34,35,55,56,57], involve co-cultures with stromal or immune cells [58,59,60,61,62], test for drugs response [37,63,64], and do not employ the specific agarose micro-mold culture model used in this study. This work’s novelty lies in the extensive characterization of nine breast cancer cell lines across different molecular subtypes and their correlation with the EMT status and spheroids’ morphology. Moreover, it provides a new, reliable platform for generating 3D spheroids using agarose micro-molds, providing a robust basis for studying subtype-specific biology, including responses to therapies, EMT processes, and cell-cell interactions. One of the major advantages of this model is the possibility of doing co-cultures with several types of cells, such as immune, stromal, and/or endothelial cells [24,25,65,66]. Future studies should explore longer-term cultures to assess necrotic core formation and integrate stromal or immune components to better mimic the tumor microenvironment. Additionally, investigating the mechanisms underlying discrepancies between EMT markers expression and spheroids morphology could yield insights into cell-cell adhesion, invasiveness, and metastatic potential. Overall, while cell line–derived 3D spheroids lack stromal and immune components and are cultured in serum-supplemented media, they provide a reproducible and standardized platform for comparative analyses across breast cancer models before validation in more complex systems, such as patient-derived organoids or co-culture models.

## 4. Materials and Methods

### 4.1. Breast Cancer Cell Lines

Breast cancer cell lines were obtained as follows: MCF-7/AZ, from Prof. Marc Mareel’s lab (Ghent University, Gent, Belgium); T47D, BT474, SK-BR-3, MDA-MB-231, BT-20, and MDA-MB-468, from ATCC (Manassas, VA, USA); MDA-IBC-3, kindly provided by Prof. Wendy Woodward; and SUM149PT, kindly supplied by Dr. Stephen Ethier. MCF-7/AZ was cultured in DMEM/F12 (Invitrogen, Paisley, UK), and T47D was cultured in RPMI 1640 with GlutaMAX (Gibco, Waltham, MA, USA). Both media were supplemented with 10% FBS (Cytiva, Marlborough, MA, USA) and 1% Pen-Strep (Invitrogen). BT474 was cultured in DMEM/F12 supplemented with 10% FBS, 2 mM L-Glutamine (Sigma Aldrich, St Louis, MO, USA), 5 µg/mL Insulin (Sigma-Aldrich), and 1% Pen-Strep. SK-BR-3, MDA-MB-231, BT-20, and MDA-MB-468 were cultured on DMEM with 10% FBS and 1% Pen-Strep. MDA-IBC-3 was cultured in Ham’s F12, with 10% FBS, 1 µL/mL Hydrocortisone (Sigma Aldrich), 5 µg/mL Insulin, 1% Pen-Strep, and 1 M HEPES (Sigma Aldrich). SUM149PT was cultured on DMEM/F12 with 5% FBS, 1 µg/mL Hydrocortisone, 5 µg/mL Insulin, and 1% Pen-Strep. All cell lines were maintained at 37 °C in a humidified atmosphere containing 5% CO_2_.

### 4.2. Breast Cancer Tumor Spheroids

Breast cancer tumor spheroids were generated through the liquid overlay technique using commercially available micro-molds (3D Petri Dish^®^, from Micro Tissues Inc., Merck, Darmstadt, Germany) (Figure S1) [24,25]. First, 2% (*w*/*v*) agarose (Grisp Research Solutions, Porto, Portugal) was dissolved in 0.9% (*w*/*v*) NaCl (Merck) and cast in 3D Petri Dish micro-molds to form agarose molds with 81 uniform circular microwells. Then, molds were placed in a 12-well plate and incubated with cell culture media (190 µL to the molds and 1 mL to the wells) to equilibrate the molds for at least 2 h. Afterwards, cells were collected and counted to prepare an adequate cell suspension, according to the three different total numbers of cells per spheroid tested (1000, 2500, and 5000 cells per spheroid). Finally, the medium was removed, and the cell suspension (190 µL) from each breast cancer cell line was added to the mold and incubated for 30 min. Finally, cell culture medium (2 mL) was added to each well.

### 4.3. Breast Cancer Spheroids’ Size Measurement

Brightfield images were acquired using a DMi1 inverted microscope (Leica, Wetzlar, Germany) at 48 h post-seeding. At the same time point, additional images were acquired using the Operetta CLS High-content Imaging System (Revivity, PerkinElmer, Waltham, MA, USA), and spheroids’ size and circularity were determined using the Harmony-High-Content Imaging and Analysis Software version 5.1. (Revivity, PerkinElmer).

### 4.4. Breast Cancer Spheroids’ Histological Analysis

After 48 h of incubation time, culture media were gently removed from the wells, and molds were washed twice with PBS. Spheroids were then fixed with 4% (*v*/*v*) of formaldehyde for 20 min at RT. Then, the formaldehyde was removed, and two more washing steps with PBS were performed. After, 1.5% agarose (*w*/*v*) solution was added to the top of each mold and allowed to solidify. Later, molds were embedded in paraffin using an automated embedding system (Thermo Scientific STP 120 Spin Tissue Processor, Waltham, MA, USA). Paraffin-embedded samples were sectioned into 4 µm sections, deparaffinized in xylene, and rehydrated through a graded alcohol series. H&E staining was performed to assess spheroid morphology. Images were acquired using the Brightfield Microscope Leica DM2000 LED (Leica).

### 4.5. Breast Cancer Spheroids’ Immunohistochemistry

Immunohistochemistry staining was carried out after 48 h of culture in conditions of 2500 cells/spheroid. Initially, samples were fixed in accordance with the protocol described above, followed by sectioning, deparaffinization in Clear-Rite 3 (Epredia, Kalamazoo, MI, USA), and rehydration through a grade series of decreasing ethanol concentrations. Thereafter, antigen retrieval was performed by incubating the sections with the appropriate antigen retrieval buffer—either sodium citrate (pH = 6) (Invitrogen) or Tris/EDTA (pH = 9) (Novocastra, Leica Biosystems, Nussloch, Germany)—for 30 min at 96 °C. After this incubation period, sections were washed twice with PBS for 5 min under gentle agitation. Endogenous peroxidase activity was blocked using 3% hydrogen peroxide (Sigma-Aldrich) in 100% methanol (Fisher Scientific, Loughborough, UK), and the sections were again washed twice with PBS. Primary antibodies were used at the dilutions presented in the Supplementary Table S1 to evaluate the expression of ER, PR, HER2 and Ki-67. Primary antibody suspension was added to each section and incubated overnight at 4 °C in a humidified chamber. Subsequently, samples were washed twice with PBS under the conditions described above. To detect the primary antibody, sections were incubated in HRP polymer (Dako, Agilent Technologies, Glostrup, Denmark) for 30 min. After three washes with PBS, 100 µL of DAB reagent (Dako) per mold was added to each section for 5 min to visualize the antibody staining. The sections were washed under running tap water twice, for 3 min each. Counterstaining was performed by immersing the sections in hematoxylin (Epredia) for 30 s to 2 min, followed by rinsing under running tap water for 5 min. Finally, tissues were dehydrated in three increasing concentrations of alcohol (70%, 100%, and 100%), each for 5 min, and cleared with Clear Rite twice for 10 min each. Slides were mounted with coverslips using a mounting solution (Richard-Allan Scientific, Thermo Fisher Scientific, Kalamazoo, MI, USA). Images were acquired using the Brightfield Microscope Leica DM2000 LED (Leica).

### 4.6. Cell Viability

A fluorescent-based staining protocol was employed, using Calcein-AM, PI, and Hoechst 3342, to assess cell viability within spheroids. Staining solutions were prepared according to Kessel et al. 2017 [67], using the following final concentrations: calcein (1 µM, Invitrogen, Thermo Fisher Scientific, C1430), PI (4 µg/mL, Invitrogen, Thermo Fisher Scientific, P1304MP), and Hoechst (10 mg/mL, Invitrogen, Thermo Fisher Scientific, H3570). After 48 h, spheroids were washed twice with PBS, and 200 µL of the staining solution was added to each well. The plate was incubated at room temperature for 45 min with gentle agitation (60 rpm). Following the incubation period, spheroids were washed again with PBS, and 200 µL of fresh PBS was added to each well. Images were acquired using the Operetta CLS high-content imaging system (Revivity). The fluorescence signals corresponded to live (green), dead (red), and total (blue) cell populations. Fluorescence intensities of calcein and PI were quantified using the Harmony High-Content Imaging and Analysis Software version 5.1 (Revivity, PerkinElmer). The software also calculated the calcein/PI fluorescence intensity ratio, which was used as an indicator of cell viability.

### 4.7. Immunofluorescence

Immunofluorescence staining was carried out after 48 h of culture in 2500 cells/spheroid for the EMT and cell death biomarkers: E-cadherin, P-cadherin, vimentin, and cleaved-Caspase 3 (cCasp3). First, sections were deparaffinized in Clear-Rite (Epredia), followed by rehydration through a series of decreasing ethanol concentrations. Thereafter, antigen retrieval was performed as described above. Subsequently, samples were washed twice with PBS for 5 min each under gentle agitation (60 rpm). To block non-specific binding, sections were incubated with 5% (*v*/*v*) BSA (NZyTech, Lisbon, Portugal) for 30 min. Primary antibodies, at the dilutions specified in Supplementary Table S1, were then applied and incubated overnight at 4 °C in a humidified chamber. Following this, sections were washed three times with PBS and incubated for 1 h in the dark with the appropriate secondary antibodies diluted 1:500 in PBS: Alexa Fluor 488 Goat Anti-Mouse IgG and Alexa Fluor 594 Goat Anti-Rabbit IgG (Thermo Fisher Scientific). After three additional washes with PBS, sections were mounted using Vectashield containing DAPI (5 µL/section; Vector Laboratories, Newark, CA, USA) and imaged with a Zeiss Axio Imager fluorescence microscope (Carl Zeiss AG, Oberkochen, Germany).

### 4.8. Bioinformatics Analysis

#### 4.8.1. CCLE Dataset

Normalized gene expression data for the expression of 53,971 genes in different breast cancer cell lines was retrieved from the Cancer Cell Line Encyclopedia (CCLE, https://depmap.org/portal/ [accessed on 11 March 2026]). Only the data from the cell lines of interest for this study (BT474, MCF-7/AZ, T47D, BT-20, MDA-MB-468, MDA-MB-231, SK-BR3, SUM149PT) were used for analysis; data from MDA-IBC3 was not available. Cell line annotations, including DepMap IDs and stripped cell line names were obtained from the CCLE cell line metadata file (sample_info.csv). The dataset was filtered to include only breast cancer lines with complete expression and annotation data. Expression values were log2-transformed RNA-seq TPM levels, as provided by CCLE.

#### 4.8.2. EMT Gene Selection

Hallmark Epithelial-Mesenchymal Transition (EMT) genes were retrieved from the Molecular Signatures Database using the msigdbr R package. A total of 200 genes associated with EMT were used to evaluate the mesenchymal characteristics of each cell line. Genes were matched to CCLE expression data based on HGNC symbols, extracting only columns corresponding to these EMT genes.

#### 4.8.3. EMT Score Calculation

For each cell line, an EMT score was computed as the mean expression across all 200 EMT genes. Missing values were ignored in the calculation. This score provides a quantitative measure of the mesenchymal phenotype of each cell line.

#### 4.8.4. Data Visualization

EMT scores were visualized using boxplots with the ggplot2 R package. Points were colored black and labeled with cell line names using the ggrepel package to avoid overlapping labels. Statistical comparisons between compact and loose spheroid groups were performed using an unpaired two-sided *t*-test (ggpubr package), with *p*-values reported on the plot.

#### 4.8.5. Data Processing

All data processing, filtering, and analyses were performed in R version 4.4.4 (R Foundation for Statistical Computing, Vienna, Austria).

### 4.9. Statistical Analysis

Statistical analyses were performed to compare experimental groups based on initial cell seeding densities (1000, 2500, and 5000 cells per spheroid) in at least three independent experiments. All statistical analyses were conducted using GraphPad Prism version 10.02 (GraphPad Software). If the assumptions for parametric analysis were met, the statistical significance was analysed in one-way analysis of variance (ANOVA) to compare differences between groups. When only the assumption of normality was satisfied, We’ch’s ANOVA was applied to account for unequal variances. However, if both normality and homogeneity of variances were confirmed, the standard one-way ANOVA was performed. If the assumptions were not satisfied, the non-parametric Kruskal–Wallis test was used instead. The level of significance was set at * *p* < 0.05, ** *p* < 0.01, *** *p* < 0.001. **** *p* < 0.0001 and ns, not significant (*p* ≥ 0.05).

## Figures and Tables

**Table 1:** Breast cancer cell lines used in this study, with their clinical origin, source and molecular tumor features.

				Molecular Subtype	
Cell Line	Disease	Source	Molecular Markers	Neve et al. [22]	Prat A et al. [14,23]
MCF-7/AZ	Invasive breast carcinoma	Pleural Effusion	ER+/PR+/HER2-	Luminal	Luminal
T47D	Invasive breast carcinoma	Pleural Effusion	ER+/PR+/HER2-		
BT474	Invasive breast carcinoma	Primary tumor	ER+/PR+/HER2+		HER2-enriched
SK-BR-3	Breast adenocarcinoma	Pleural effusion	ER-/PR-/HER2+		HER2-enriched
MDA-IBC-3	Breast inflammatory carcinoma	Pleural effusion	ER-/PR-/HER2+	-	HER2-enriched
BT20	Invasive breast carcinoma	Primary tumor	ER-/PR-/HER2-	Basal A	Basal
MDA-MB-468	Breast adenocarcinoma	Pleural effusion	ER-/PR-/HER2-	Basal A	Basal
SUM149PT	Breast inflammatory carcinoma	Primary tumor	ER-/PR-/HER2-	Basal B	Basal/Claudin-low
MDA-MB-231	Breast adenocarcinoma	Pleural effusion	ER-/PR-/HER2-	Basal B	Claudin-low

## Data Availability

All data generated or analyzed during this study are included in this published article and its Supplementary Information files.

## Supplementary Materials

The following supporting information can be downloaded at https://www.mdpi.com/article/10.3390/ijms27083529/s1.
