# Supplementary material for: From Luminal to Triple Negative: 3D Spheroids Reveal Molecular and Phenotypic Differences Across Breast Cancer Subtypes
Source: Int J Mol Sci. 2026 Apr 15;27(8):3529. doi: 10.3390/ijms27083529 (PMC13116449; doi:10.3390/ijms27083529)
Supplement: Supplementary file 1 [file ijms-27-03529-s001.zip › ijms-4129652-supplementary.pdf]

Supplementary Materials

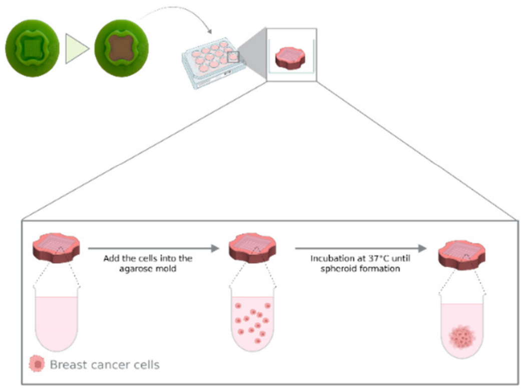

Figure S1: Graphic illustration of breast cancer tumor spheroids formation using the liquid overlay technique. Figure created in BioRender. Castro, MM (2026) https://BioRender.com/ bdfe1y0 (accessed on 9th April of 2026).

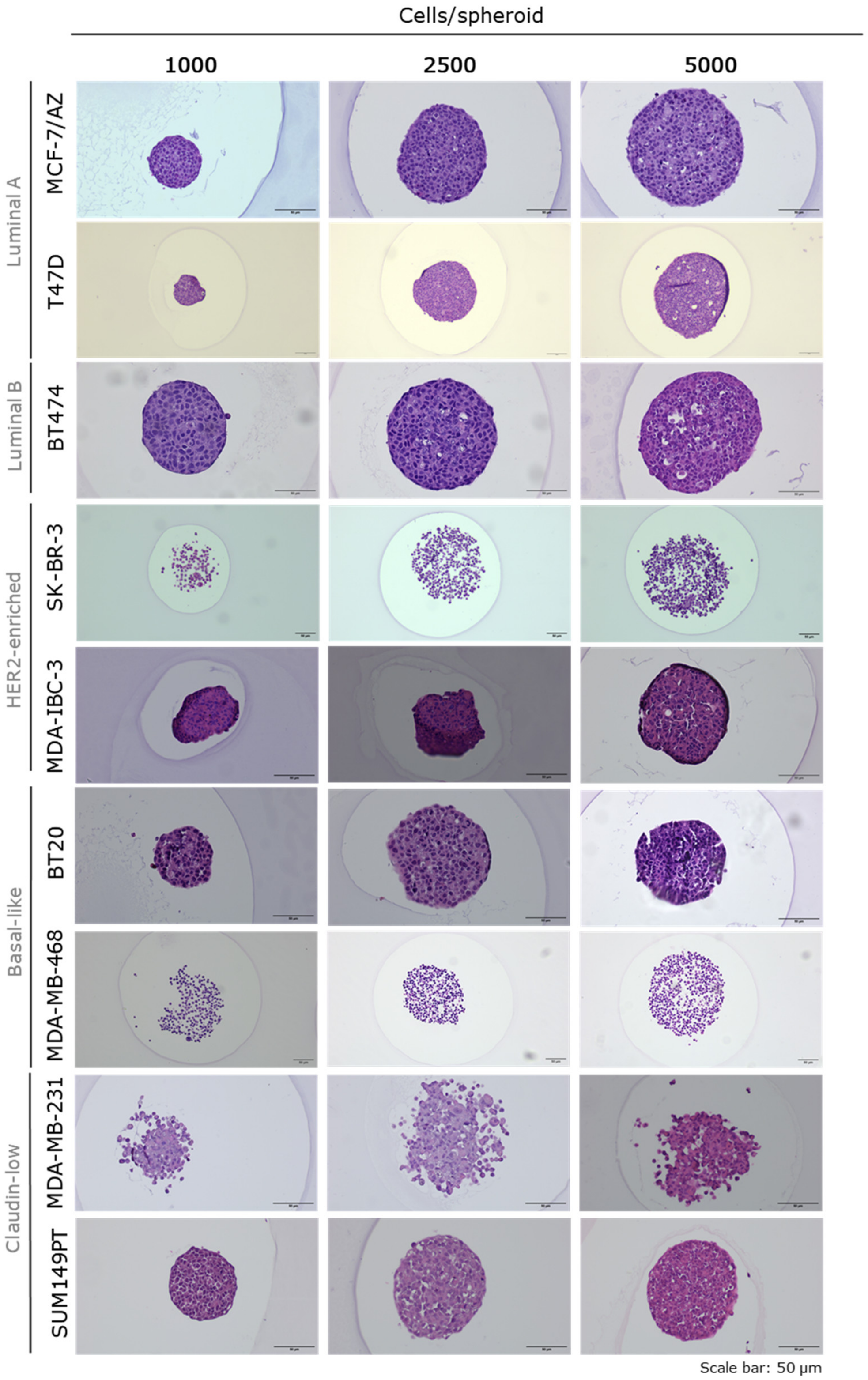

Figure S2: Histological analysis by H&E staining of breast cancer spheroids after 48h grown in suspension in three cell densities. Scale bars represent 50 µm.

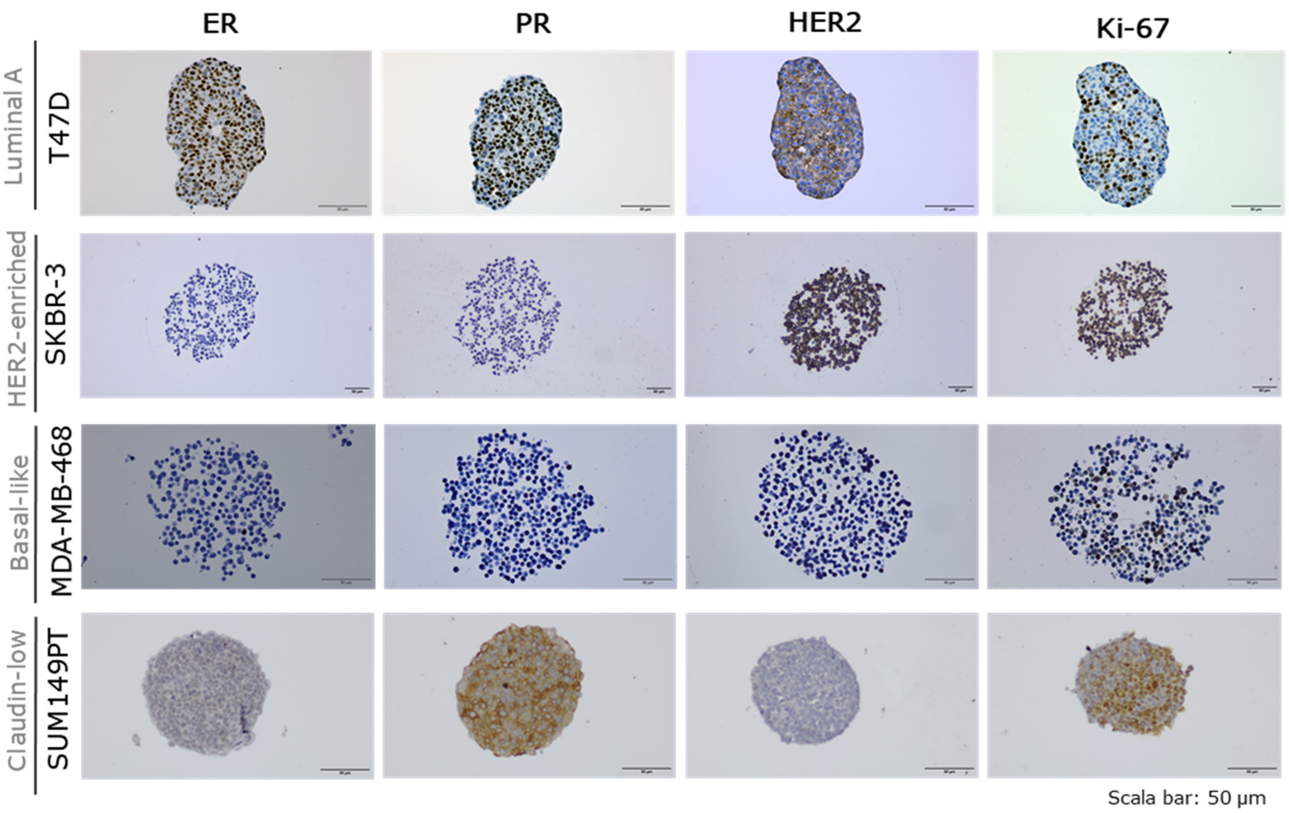

Figure S3: Representative images of breast cancer spheroids from distinct molecular subtypes (Luminal A-T47D, HER2-enriched–SKBR-3, Basal-like-MDA-MB-468, and Claudin-low–SUM149PT). Immunohistochemistry for the Hormonal receptors (ER-Estrogen; PR-Progesterone), human epidermal growth factor receptor 2 (HER-2) and Ki67 (proliferation marker) using breast cancer spheroids (2500 cells/spheroid). Scale bars represent 50 µm.

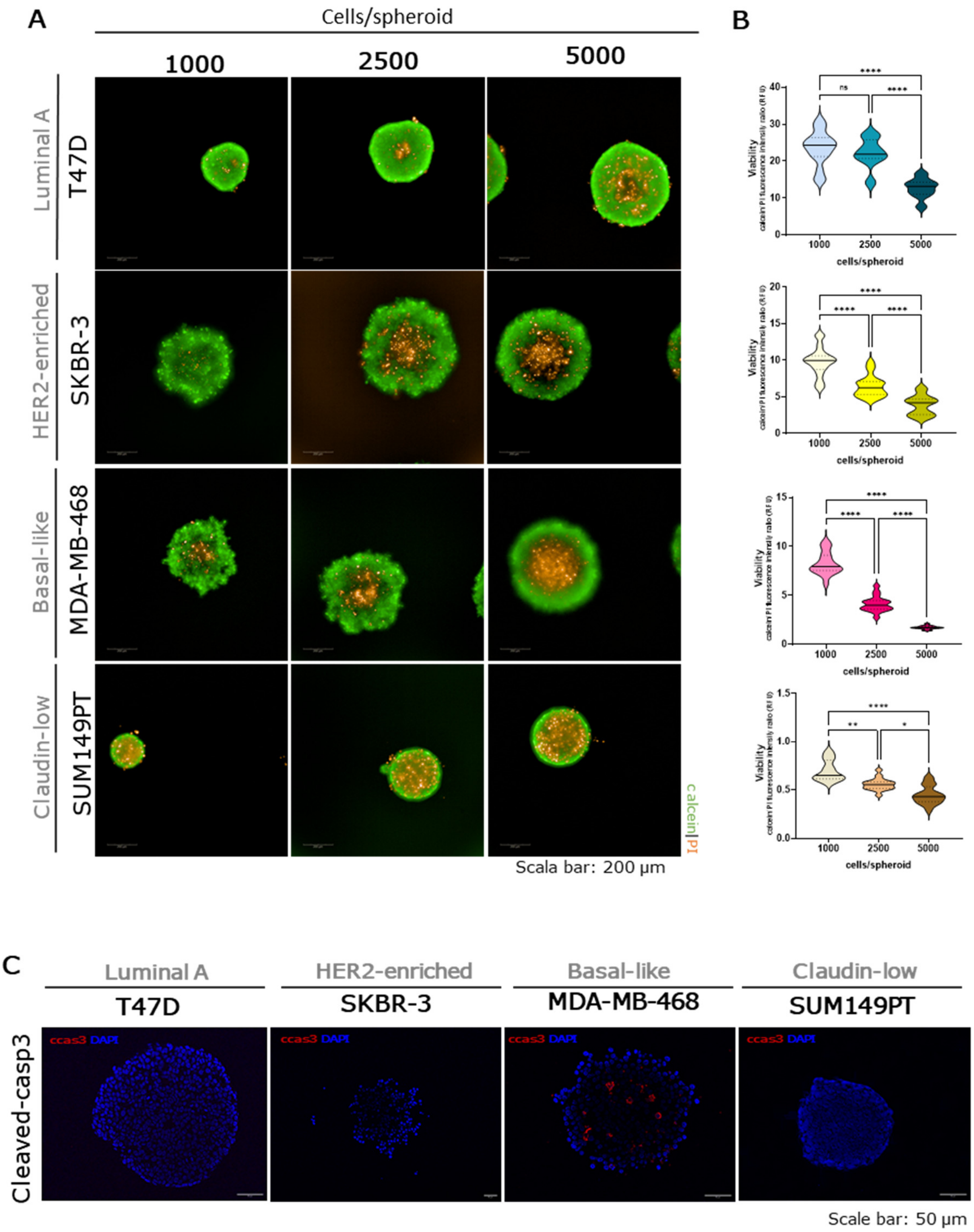

Figure S4: Evaluation of cell viability and cleaved-caspase 3 of breast cancer spheroids from distinct molecular subtypes: Luminal A-T47D (blue), HER2-enriched–SKBR-3 (yellow), Basal-like-MDA-MB-468 (pink), and Claudin-low–SUM149PT (brown). Viability was evaluated using calcein/PI staining, where calcein (green) label live cells, and PI (red) the dead cells. Scale bars represent 200 µm (A). Quantification of the fluorescent intensity (RFU -relative fluorescent units) of calcein and PI ratio using the Harmony high-content imaging and analysis software in three independent experiments (B). Representative images of cleaved-caspase-3 (cCasp3, red) staining and nuclei counterstained with DAPI (blue) in the 2500 cells/spheroid after 48h in suspension. Scale bars represent 50 µm (C). Data are shown as mean ± SD, and the level of significance was set at *p<0.05, **p<0.01, ***p<0.001, ****p<0.0001, and ns, not significant (p≥ 0.05).

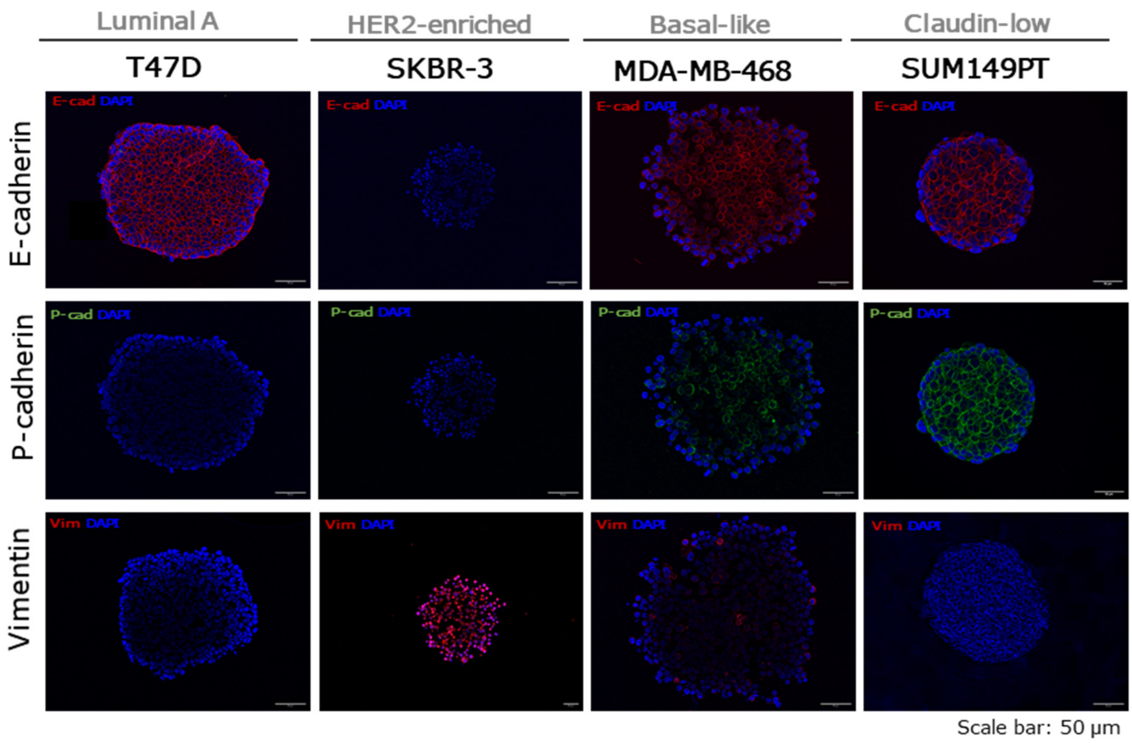

Figure S5: Representative images showing the expression of EMT markers in breast cancer spheroids from distinct molecular subtypes (Luminal A-T47D, HER2-enriched–SKBR-3, Basal-like-MDA-MB-468, and Claudin-low–SUM149PT). E-cadherin staining (red) was used as an epithelial marker, Vimentin staining (red) for mesenchymal marker, and E-cadherin (red) and P-cadherin (green) represent the hybrid EMT phenotype. All samples were counterstained with DAPI (blue). Scale bars represent 50 µm.

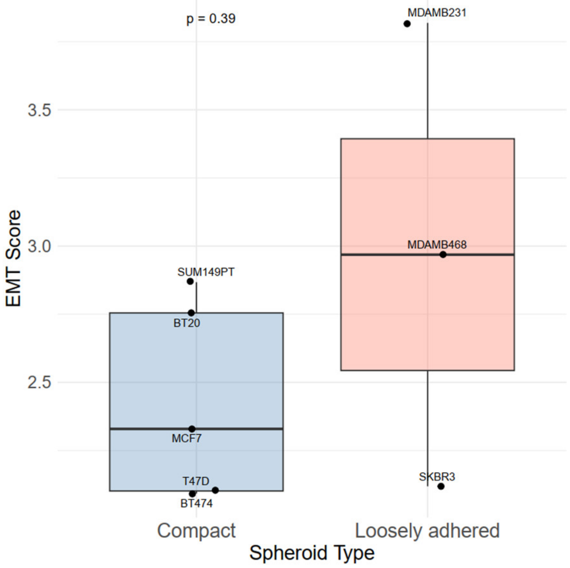

Figure S6: Boxplot showing the correlation between the EMT score and 3D spheroids' morphology: compact (MCF-7/AZ, T47D, BT474, BT-20, SUM149PT) or loosely adhered (SKBR-3, MDA-MB-468, MDA-MB-231). Graph and analysis performed in R software version 4.4.4.

Table S1: Antibodies dilutions used for protein detection by immunohistochemistry and immunofluorescence.

Immunohistochemistry				
Protein	ER	PR	HER2	Ki67
Reference/Company	NCL-L-ER-6F11, Leica Biosystems (Nussloch, Germany)	NCL-L-PGR, Leica Biosystems (Nussloch, Germany)	Clone ab134182, Abcam (Cambridge, UK)	Clone MIB-1, Dako (Glostrup, Denmark)
Dilution	1:50	1:100	1:500	1:250
Immunofluorescence				
Protein	cCasp3 (asp175) (D3E9)	E-cadherin	P-cadherin	Vimentin
Reference/Company	9579S, Cell Signaling (Danvers, MA, USA)	24E10, Cell Signaling (Danvers, MA, USA)	Clone 56, BD Transduction (East Rutherford, NJ USA)	D21H3, Cell Signaling (Danvers, MA, USA)
Dilution	1:200	1:100	1:100	1:400
